# *APOE* ε4-dependent effects on the early amyloid pathology in induced neurons of patients with Alzheimer’s disease

**DOI:** 10.1186/s40035-022-00319-9

**Published:** 2022-10-25

**Authors:** Hongwon Kim, Siyoung Kim, Byounggook Cho, Jaein Shin, Jongpil Kim

**Affiliations:** 1 Department of Biomedical Engineering, Dongguk University, Pildong-ro 1-gil 30, Jung-Gu, Seoul, 04620 Republic of Korea; 2 Laboratory of Stem Cells & Gene Editing, Department of Chemistry, Dongguk University, Pildong-ro 1-gil 30, Jung-Gu, Seoul, 04620 Republic of Korea

**Keywords:** Alzheimer’s disease, Direct conversion, Apolipoprotein E, Induced neuron, Amyloid, Presenilin

## Abstract

**Background:**

The ε4 allele of apolipoprotein E (*APOE* ε4) is the strongest known genetic risk factor for late-onset Alzheimer’s disease (AD), associated with amyloid pathogenesis. However, it is not clear how *APOE* ε4 accelerates amyloid-beta (Aβ) deposition during the seeding stage of amyloid development in AD patient neurons.

**Methods:**

AD patient induced neurons (iNs) with an *APOE* ε4 inducible system were prepared from skin fibroblasts of AD patients. Transcriptome analysis was performed using RNA isolated from the AD patient iNs expressing *APOE* ε4 at amyloid-seeding and amyloid-aggregation stages. Knockdown of IGFBP3 was applied in the iNs to investigate the role of IGFBP3 in the *APOE* ε4-mediated amyloidosis.

**Results:**

We optimized amyloid seeding stage in the iNs of AD patients that transiently expressed *APOE* ε4. Remarkably, we demonstrated that Aβ  pathology was aggravated by the induction of *APOE* ε4 gene expression at the amyloid early-seeding stage in the iNs of AD patients. Moreover, transcriptome analysis in the early-seeding stage revealed that IGFBP3 was functionally important in the molecular pathology of *APOE* ε4-associated AD.

**Conclusions:**

Our findings suggest that the presence of *APOE* ε4 at the early Aβ-seeding stage in patient iNs is critical for aggravation of sporadic AD pathology. These results provide insights into the importance of *APOE* ε4 expression for the progression and pathogenesis of sporadic AD.

**Supplementary Information:**

The online version contains supplementary material available at 10.1186/s40035-022-00319-9.

## Background

Alzheimer’s disease (AD) is an age-dependent neurodegenerative disorder, characterized by cognitive decline and memory loss [1, 2]. The major pathological hallmarks of AD are the accumulation of amyloid-β (Aβ), a product from amyloid precursor protein (APP) cleavage, and neurofibrillary tangles composed of phosphorylated tau protein [3, 4]. Previous studies have indicated that Aβ begins to deposit in the brains of AD patients much earlier than the onset of clinical symptoms [5, 6]. Therefore, the initiating step, in which small aggregates begin to form, offers a time window to prevent aggravation of AD pathology [7].

The ε4 allele of apolipoprotein E gene (*APOE* ε4) is a major risk factor for sporadic AD; it is associated with fibrillary Aβ burden and promotes Aβ aggregation in late-onset AD [8–11]. Recent studies have reported that the expression of *apoE* ε4 during the initial seeding stage of plaque formation is sufficient to drive amyloid pathology and dystrophic neurites in amyloid model mice [12]. Moreover, Huynh et al. [13] showed that reducing *apoE* levels prior to plaque deposition critically affects plaque formation in APP/PS1-21 mice harboring a homozygous *apoE* ε4 allele. However, these phenotypes have mostly been shown in the context of murine models with *apoE* ε4 overexpression. Thus, it is necessary to determine the effects of *APOE* ε4 on plaque formation in appropriate human neurons. Moreover, not much is known about the molecular targets that mediate the effects of *APOE* ε4 on plaque formation, which is required for disease progression in the brains of AD patients.

Recent pioneering works on neuronal reprogramming have established the feasibility of direct conversion of human somatic cells into functional neurons [14–17], which can ultimately be applied to model neurological disorders and understand novel pathogenic mechanisms. Moreover, iNs derived from human somatic cells with AD-associated mutations or risk alleles are promising in vitro models for disease phenotypes and pathological changes [18, 19]. Hence, iNs of AD patients (hereafter denoted as “AD patient-derived iNs”) with a human *APOE* ε4 background would provide a useful approach to understanding the pathogenesis of *APOE* ε4-mediated sporadic AD and facilitating further therapeutic discovery.

In this study, we set out to examine whether *APOE* ε4 aggravates amyloid pathogenesis as per the stage of amyloid seeding in human iNs, and then analyze the transcriptional regulatory network to identify factors that mediate the effect of *APOE* ε4 at the amyloid early-seeding stage. The aim of this study was to demonstrate the cellular and molecular mechanisms of the effects of *APOE* ε4 expression at the amyloid-seeding stage on AD pathogenesis in AD patient iNs and provide a functional candidate for therapeutic modalities.

## Methods

### Culture of human fibroblasts

Human fibroblasts were cultured in a human fibroblast medium (DMEM medium containing 10% fetal bovine serum, 1% nonessential amino acid (Gibco, Waltham, MA), 0.1% β-mercaptoethanol (Gibco), and 1% penicillin/streptomycin (Gibco)). Human control (GM23967, male, condition: healthy control, *APOE* ε3/3 genotype) and AD fibroblasts (AG06848, female, condition: AD *PSEN1 (ALA246GLU)* mutation, *APOE* ε3/3 genotype; AG09908, female, condition: AD *PSEN2 (ASN141ILE)* mutation, *APOE* ε3/3 genotype; AG05810, female, condition: *APOE* ε3/4 genotype; AG04402, male, condition: *APOE* ε3/4 genotype; AG05770, male, condition: unknown, *APOE* ε3/3 genotype) were purchased from the Coriell Cell Repository (Camden, NJ).

### Direct conversion of human fibroblasts into iNs

HEK293T cells were transfected with the lentivirus construct, Ascl1, Brn2, Myt1l, M2rtTA, APOE ε3, APOE ε4, psPAX2 and pMD2.G vectors through calcium phosphate co-precipitation. A previously published protocol [20] was used to generate the lentivirus from the transfected HEK293T cells. To generate human iNs, human fibroblasts were infected with the lentivirus (FUW-Ascl1, Brn2, Myt1l) 3 times in 2 days. After approximately 48–72 h of infection, the medium was replaced with the N3 medium containing DMEM/F12, insulin (25 µg/ml), progesterone (20 nM), transferrin (50 µg/ml), putrescine (100 µM), laminin (1 µg/ml), FGF basic (25 µg/ml), BDNF (10 g/ml), Forskolin (5 µM), and 1% penicillin/streptomycin. Additionally, for *APOE* ε3 or *APOE* ε4 induction in human iNs, doxycycline (2 µg/ml) was added at 7 or 14 days after lentivirus (FUW-Ascl1, Brn2, Myt1l) infection.

### Immunofluorescence analysis

The cultured AD patient iNs were washed with  1× phosphate-buffered saline (PBS), fixed in 4% paraformaldehyde and washed twice with 1× PBS containing 0.1% triton-X. Primary antibodies (anti-βIII-tubulin, 1:1000, Sigma-Aldrich, St. Louis, MO; NeuN, 1:500, Millipore, Darmstadt, Germany; MAP2, 1:200, Cell Signaling, Beverly, MA; VGLUT1, 1:200, Invitrogen, Grand Island, NY; Synapsin 1, 1:500, Invitrogen; Aβ (6E10), 1:500, Biolegend, San Diego, CA; Aβ42, 1:500, Biolegend; Phosphorylated tau, 1:400, Pierce, Rockford, IL;  apoE4, 1:500, Millipore; LC3B, 1:500, Cell Signaling; EEA1, 1:500, Millipore) were applied overnight at 4 °C. Appropriate secondary antibodies were obtained from Invitrogen and incubated for 2 h at room temperature. After washing, the samples were treated with 6-diamidino-2-phenylindole (DAPI, Invitrogen) and mounted in Fluoromount-G mounting medium. Representative images were taken on a Zeiss confocal microscope (Zeiss, Oberkochen, Germany, LSM800). An investigator blinded to the experimental conditions analyzed all tests. Image J software was used to analyze particles and to quantify immunofluorescent signals within regions of interest. These data were processed in parallel on the same confocal microscope with the same setting.

### Western blot analysis

Samples of AD-patient iNs were washed with 1 × PBS and then lysed in RIPA buffer containing 1% NP-40, 0.5% DOC, 0.1% SDS, and 150 mmol/l NaCl in 50 mmol/l Tris (pH 8.0) supplemented with 1 × proteinase inhibitor mixture (GenDepot, Barker, TX). Following the previously published protocol [21], the supernatant for Aβ analysis was electrophoresed on 12% sodium dodecyl sulfate–polyacrylamide gel and transferred to nitrocellulose membranes (GE Healthcare Bio-Sciences, Piscataway, NJ). Primary antibodies (anti-Aβ (6E10), 1:300, Biolegend; apoE4, 1:1000, Millipore; IGFBP3, 1:1000, Santa Cruz, Dallas, TX; β-actin, 1:1000, AbFrontier, Seoul, Korea) were applied overnight at 4 °C. Representative images were obtained using Chemidoc TRS + with Image Lab software (Bio-Rad Laboratories, Hercules, CA).

### Flow cytometry

Cells were detached using 0.125% trypsin–EDTA for 4 min. Single cells were incubated in 4% paraformaldehyde for 10 min at 4 °C. The cells were washed twice with 1% bovine serum albumin. After washing, the cells were resuspended in fluorescence-activated cell sorting buffer and filtered using a 40-µm cell strainer for analysis. Flow cytometry was performed using an Accuri instrument (Becton, Dickinson and Company, San Jose, CA). FAC gates were set for Synapsin-red fluorescent protein (RFP) + cells compared to the control level. Quantification of individual data was performed with FlowJo vX software (TreeStar, Ashland, OR).

### Aβ ELISA

ApoE3- or apoE4-expressing iNs were cultured for 3 weeks and the culture medium was replaced with fresh neuronal culture media. After 72–96 h, the neuronal culture media were collected. To extract intracellular Aβ40 and Aβ42, the cells were lysed with ELISA sampling buffer ( 1× TBS buffer, 1% Triton X-100, 0.1% SDS), followed by centrifugation at 20,000* g* for 30 min. The collected samples were assayed for  Aβ42 and Aβ40 using ELISA kits (human amyloid-beta assay ELISA kits, IBL, Hamburg, Germany), according to the manufacturer’s protocols.

### Quantitative RT-PCR analysis

The detailed procedures were as previously described [22]. qRT-PCR analysis was conducted by 1/50 of the reverse transcription reaction in a Rotor-Gene Q (QIAGEN, Hilden, Germany). The gene expression of each marker was normalized against GAPDH in each sample. The following gene primers were employed: *APOE* forward: 5′-AGG CCA AGG TGG AGC AAG-3′, reverse: 5′-CCT GCA CCT GCT CAG ACA G-3′; *IGFBP3* forward: 5′-AAA TGC TAG TGA GTC GGA GG-3′, reverse: 5′-CTG GGT ATC TGT GCT CTG AG-3′.

### Gene expression profiling using microarray

cDNA was synthesized using the GeneChip WT (Whole Transcript) Amplification kit according to the manufacturer’s protocol. One sample from each condition was prepared. Affymetrix GeneChip Human Gene 2.0 ST Array was performed according to the manufacturer’s protocol. Robust multiarray averaging (RMA) method with the affy R package was used for normalization and summarization. When multiple probes were available, average values per gene were applied.

### Gene set enrichment (GSEA) and network analysis

GSEA was performed using the GSEA preranked mode to determine whether gene sets [23] were statistically enriched in both *APOE* ε4-expressing AD patient iNs (Dox, 7–20 on) versus AD-patient iNs and *APOE* ε4-expressing AD-patient iNs (Dox, 14–20 on) versus AD patient iNs in the human brain tissue (GSE48350). Curated gene sets (1672 genes of BLALOCK_ALZHEIMERS_DISEASE) in Molecular Signatures Database (MSigDB) v5.1 and differentially expressed genes (DEGs) (FC ≥ 1.5) in *APOE* ε4-expressing AD patient iNs/patient iNs were used. The results of GSEA were considered significant when FDR and nominal *P*-value were less than 0.05. The interactome of proteins in *Homo sapiens* were obtained from STRING (https://string-db.org/, v11.5). To identify the network between AD and the IGFBP3 gene, protein–protein interactions among the DEGs of *APOE* ε4-expressing AD-patient iNs (+ APOE4, day 7) and gene set of the AD brain tissue were processed in Cytoscape (http://www.Cytoscape.org, v3.9.1).

### Statistical analysis

Data are presented as mean ± SEM of each independent experiment. *n* indicates the number of individual experiments. Dots represent the number of independent experiments. The experiments were performed with at least three independent technical replicates. Analysis of variance (ANOVA) test was used for multicomponent comparisons and Student's *t*-test for two-component comparisons after normal distribution was confirmed. ANOVA followed by Tukey–Kramer multiple comparison tests was performed with GraphPad Prism. All statistical details of the experiments are presented in figure legends.

## Results

### *APOE* ε4-expressing iNs from patients with AD

**Fig. 1:**
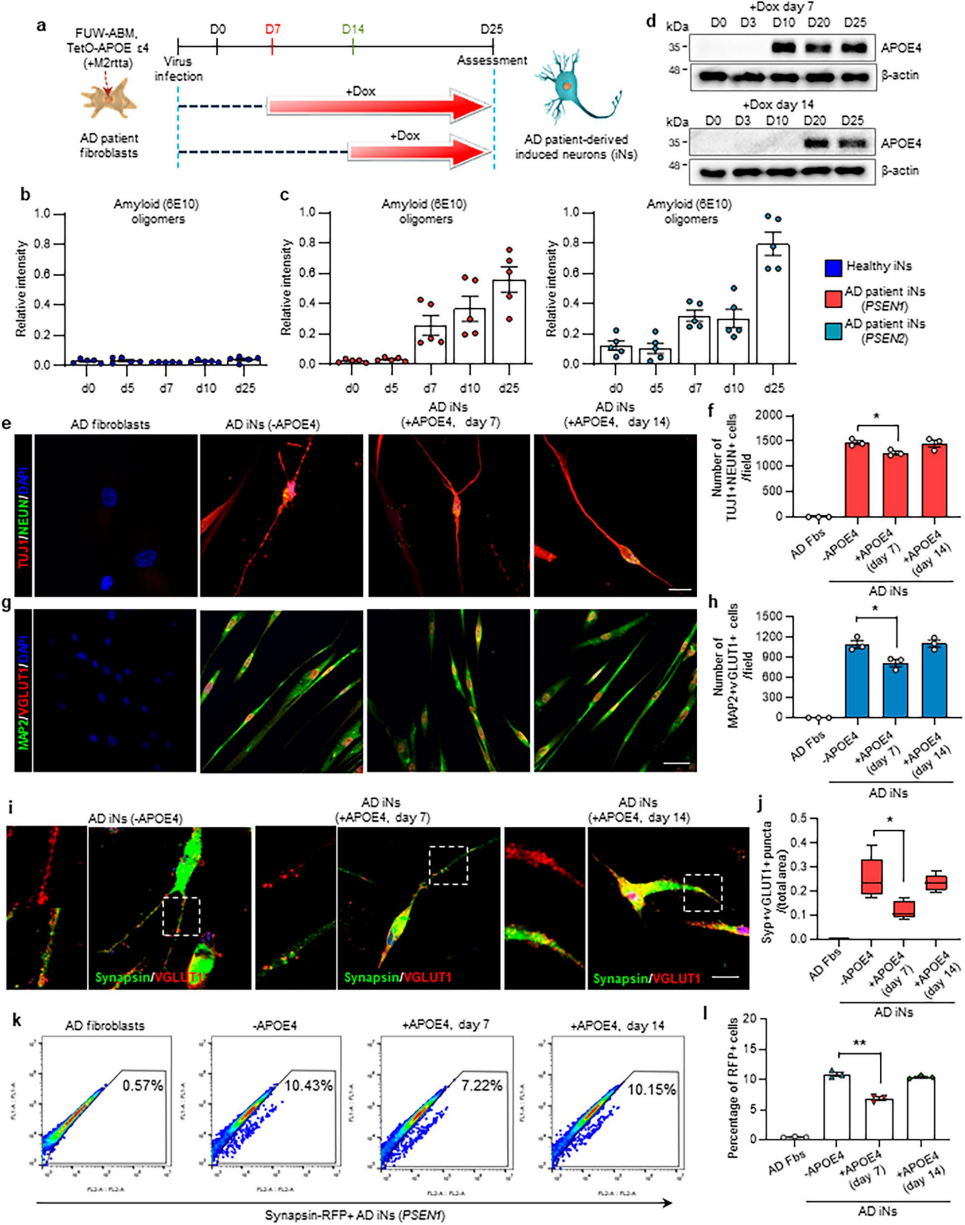
*APOE* ε4-expressing induced neurons (iNs) from patients with Alzheimer’s disease (AD). **a** Schematic depiction of the generation of directly converted iNs from the fibroblasts of patients with AD. To induce APOE ε4 isoforms at the time point of the amyloid-seeding stage, doxycycline was added at day 7 (amyloid-seeding stage) and 14 (amyloid-progressive stage). **b** Relative intensity of  Aβ (6E10) oligomers in the iNs of healthy controls at different time points. Data represent mean ± SEM. ANOVA-test; *n* = 5 per sample. **c** Relative intensity of amyloid oligomers (6E10) in AD patient iNs harboring *PSEN1* (left) and *PSEN2* (right) mutation at different time points. Data represent mean ± SEM. ANOVA-test, *P < 0.05, **P < 0.01; *n* = 5 per sample. **d** Western blot analysis of *APOE* ε4 in AD patient-derived iNs treated with doxycycline at different time points. **e** Immunofluorescence for TUJ1 and NEUN to confirm AD patient iNs on day 25. Scale bar,  20 µm. **f** Quantification of TUJ1^+^/NeuN^+^ cells at amyloid-seeding and amyloid-aggregation stages. Data represent mean ± SEM. ANOVA-test, *P < 0.05; *n* = 3 per sample. **g** Immunostaining of MAP2- and VGLUT1-positive cells in *APOE* ε4-expressing AD patient-derived iNs on day 25. Scale bars, 50 µm. **h** Quantification of MAP2^+^/VGLUT1^+^ cells at amyloid-seeding and amyloid-aggregation stages. Data represent mean ± SEM. ANOVA-test, *P < 0.05; *n* = 3 per sample. **i** Immunostaining of Synapsin1- and VGLUT1-positive cells in AD patient iNs. Scale bar, 20 µm. **j** Number of Synapsin^+^/VGLUT1^+^ puncta in each condition. Data represent mean ± SEM. ANOVA-test, *P < 0.05; *n* = 5 per sample. **k** Fluorescence-activated cell-sorting analysis of Synapsin–RFP-positive cells from AD fibroblasts harboring *PSEN1* mutation, AD patient iNs, and *APOE *ε4-expressing patient iNs. **l** Quantification of Synapsin–RFP-positive cells in each condition. Data represent mean ± SEM. ANOVA-test, *P < 0.05; *n* = 3 per sample. + APOE4 (day 7): AD patient iNs expressing *APOE* ε4 from day 7; + APOE4 (day 14): AD patient iNs expressing *APOE* ε4 from day 14; -APOE4: AD patient iNs with no *APOE* ε4 expression

To prepare AD patient iNs that express *APOE* ε4 at the amyloid-seeding and amyloid-aggregation stages, fibroblasts of AD patients harboring presenilin mutations were transduced with lentivirus constitutively expressing Ascl1, Brn2, and Myt1l (ABM) along with doxycycline-inducible *APOE* ε4 lentivirus (Fig. 1a). Since we were able to identify amyloid (6E10) oligomers at day 7 in AD patient iNs (Fig. 1b, c; Additional file 1: Fig. S1a-d), we conditionally overexpressed *APOE* ε4 with doxycycline from day 7 as the amyloid oligomer-seeding stage and day 14 as the amyloid-oligomer progressive stage after ABM induction (Fig. 1a). We confirmed the expression of apoE4 protein in *PSEN1* and *PSEN2* AD patient iNs after 10 and 20 days of ABM induction, which continued up to 25 days of direct conversion (Fig. 1d; Additional file 1: Fig. S1e, f).

We confirmed the expression of mature neuronal markers such as TUJ1, NeuN, MAP2, and VGLUT1 (Fig. 1e, g) in AD fibroblasts and iNs. The AD patient iNs exhibited well-developed and mature neuronal characteristics, but the number of TUJ1^+^/NeuN^+^ cells was slightly decreased in the AD patient iNs that expressed *APOE* ε4 at the amyloid-seeding stage, compared to the AD patient iNs without *APOE* ε4 expression, while no such difference was found when *APOE* ε4 was expressed at the amyloid progressive stage (Fig. 1f). Consistently, the number of MAP2^+^/VGLUT1^+^ cells was significantly decreased in the AD patient iNs that expressed *APOE* ε4 at the amyloid-seeding stage (Fig. 1g, h), indicating that the conditional expression of *APOE* ε4 at the amyloid-seeding stage may affect the degeneration of AD patient iNs.

To assess the effects of *APOE* ε4 on synaptic changes at different amyloid-seeding stages in AD iNs, we examined the expression of a presynaptic marker, VGLUT1, and a postsynaptic marker, Synapsin1, in the *APOE* ε4-expressing AD patient iNs. Importantly, reprogramming for 25 days resulted in a significant decrease in the number of Synapsin1^+^/VGLUT1^+^ puncta in the AD patient iNs with *APOE* ε4 expression from day 7, the amyloid-seeding state; in contrast, *APOE* ε4 expression after the amyloid-seeding state did not affect the number of Synapsin1^+^/VGLUT1^+^ puncta (Fig. 1i, j). Additionally, we counted RFP-positive iNs derived from AD patient iNs harboring a Synapsin-RFP reporter. Flow cytometric analysis showed that the number of Synapsin-RFP^+^  cells significantly decreased in the iNs of AD patients with *APOE* ε4 expression at the amyloid-seeding state (Fig. 1k, l; Additional file 1: Fig. S1g). These data indicate that alteration of synaptic function is possibly affected by *APOE* ε4 induction at the amyloid-seeding stage.

### Increase in AD-related phenotypes by *APOE* ε4 expression from the amyloid-seeding stage

**Fig. 2:**
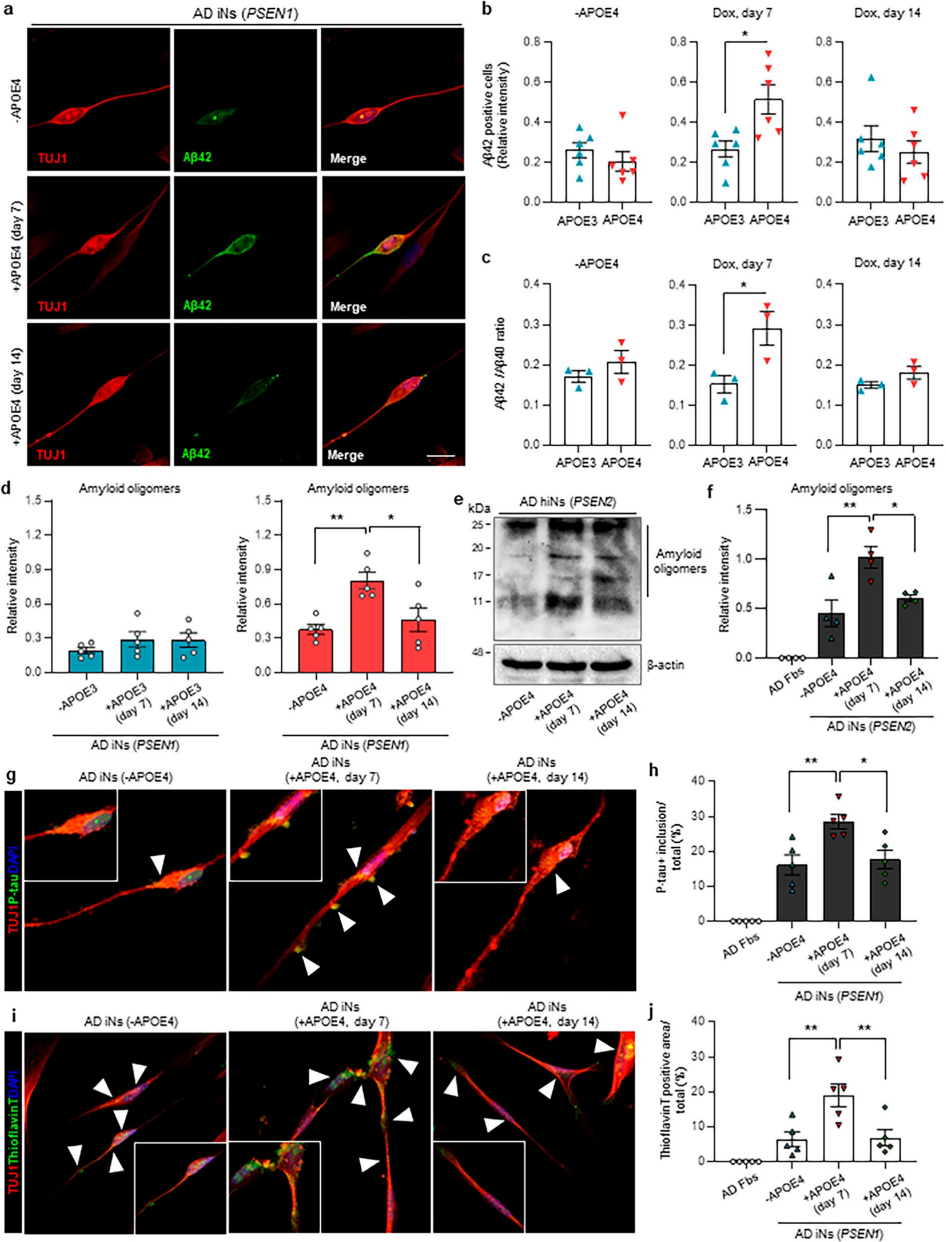
Increase in AD-related phenotypes by *APOE* ε4 expression from the amyloid-seeding stage. **a** Representative immunofluorescence images of Aβ42-positive iNs of the *PSEN1* mutation line at day 25. To express *APOE* ε4 in AD patient iNs, doxycycline was added on day 7 or 14. Scale bar, 20 µm. **b** Relative intensity of Aβ42-positive cells in *APOE* ε3- or *APOE* ε4-expressing AD patient iNs at different amyloid stages. Data represent mean ± SEM. ANOVA-test, *P < 0.05, **P < 0.01; *n* = 6 per sample. **c** Ratio of Aβ42 to Aβ40 concentration in *APOE* ε3- or *APOE* ε4-expressing AD patient iNs. Data represent mean ± SEM. ANOVA-test, *P < 0.05, **P < 0.01; *n* = 3 per sample. **d** Relative intensity of amyloid oligomers in *APOE* ε3- or *APOE* ε4-expressing AD patient iNs at different amyloid stages. Data represent mean ± SEM. ANOVA-test, *P < 0.05, **P < 0.01; *n* = 5 per sample. **e** Representative image of western blot analysis showing the level of Aβ (6E10) oligomers in *APOE* ε4-expressing AD patient iNs harboring *PSEN2* mutation at different amyloid stages. **f** Relative intensity of amyloid oligomers in *APOE* ε4-expressing AD patient iNs harboring *PSEN2* mutation. Data represent mean ± SEM. ANOVA-test, *P < 0.05, **P < 0.01; *n* = 4 per sample. **g** Representative immunofluorescence images of hyperphosphorylated tau in *APOE* ε4-expressing AD patient iNs harboring *PSEN1* mutation on day 25. Scale bar, 20 µm. **h** Measurement of phospho-tau-positive inclusions compared to the total area at different amyloid stages. Data represent mean ± SEM. ANOVA-test, *P < 0.05, **P < 0.01; *n* = 5 per sample. **i** Representative images of thioflavin T staining in *APOE* ε4-expressing AD patient iNs harboring *PSEN1* mutation at different amyloid stages. White arrows indicate thioflavin T-positive area. Scale bar, 20 µm. **j** Quantification of thioflavin T-positive area in *APOE* ε4-expressing AD patient iNs. Data represent mean ± SEM. ANOVA-test, *P < 0.05, **P < 0.01; *n* = 5 per sample. + APOE4 (day 7): AD patient iNs expressing *APOE* ε4 from day 7; + APOE4 (day 14): AD patient iNs expressing *APOE* ε4 from day 14; -APOE4: AD patient iNs with no *APOE* ε4 expression

To demonstrate the effect of *APOE* ε4 expression at Aβ-seeding stages on AD-associated pathogenesis, we first examined the aggregation of Aβ42 in AD patient iNs that expressed *APOE* ε4 from the amyloid-seeding stage. We observed that the number of Aβ42^+^/TUJ1^+^ iNs significantly increased in *APOE *ε4 AD iNs that expressed *APOE* ε4 from the amyloid-seeding stage (Fig. 2a, b). However, no significant difference was observed in the relative intensity of Aβ42-expressing cells when *APOE* ε4 was expressed after the amyloid-seeding stage (Fig. 2a, b). Additionally, the Aβ42-expressing TUJ1 + iNs were not detected in healthy-control iNs that expressed *APOE* ε4 from different seeding stages (Additional file 1: Fig. S2a). A previous study showed that apoE  is co-localized with amyloid plaques in the brains of patients with AD [24]. Consistently, we found that localization of Aβ C-terminal area with apoE4 was significantly increased in AD patient iNs that expressed *APOE* ε4 from the amyloid-seeding stage (Additional file 1: Fig. S3a–c). Furthermore, abnormal localization of *APOE* ε4-positive vesicles with LC3B, a marker of autophagy, increased in the AD iNs with *APOE* ε4 expression in the amyloid-seeding stage, but not in the amyloid progressive stage (Additional file 1: Fig. S3d, e). We assessed the effect of exogenous *APOE* ε4 lipid particles in *APOE* ε3-expressing AD patient iNs. Interestingly, the number of Aβ42-expressing cells significantly increased in the amyloid-seeding stage (Additional file 1: Fig. S4a-c). Moreover, we found that *APOE* ε4 lipid particle induction at the amyloid-seeding stage dramatically increased the formation of lipid droplet in AD iNs harboring *PSEN1* mutation (Additional file 1: Fig. S4d). Additionally, *APOE* ε4 induction from the amyloid-seeding stage led to a significant increase in the Aβ42:Aβ40 ratio in the AD patient iNs (Fig. 2c). However, we did not observe a significant difference in the Aβ42:Aβ40 ratio in iNs that expressed *APOE* ε4 from the amyloid-aggregation stage (Fig. 2c).

We next sought to examine the accumulation of Aβ by *APOE* ε4 induction at the amyloid-seeding stage. First, we validated the expression of APOE 3 and 4 mRNA after doxycycline induction in AD patient iNs harboring *PSEN* mutations (Additional file 1: Fig. S5a, b). We found that *APOE* ε4 induction at the amyloid-seeding stage dramatically increased the accumulation of Aβ oligomers in AD iNs harboring *PSEN1* mutation, relative to AD iNs with *APOE* ε4 induction after the amyloid-seeding stage (Fig. 2d, Additional file 1: Fig. S5c). However, the *APOE* ε3 induction at the amyloid-seeding stage did not affect Aβ oligomers in these AD iNs (Fig. 2d). Similarly, Aβ oligomers were found to be significantly increased in AD patient iNs harboring *PSEN2* mutation with *APOE* ε4 induction at the amyloid-seeding stage (Fig. 2e, f). We also confirmed that the number of EEA1- and Aβ42-positive puncta are also increased in *APOE* ε4-expressing AD patient iNs, suggesting that *APOE* ε4 induction influences Aβ oligomeration via APP endocytosis and processing in AD patient iNs (Additional file 1: Fig. S5d). We also observed increased p-tau accumulation in cell bodies and dendrites owing to *APOE* ε4 induction at the amyloid-seeding stage (Fig. 2g, h). However, the *APOE* ε4 induction in different seeding stages in healthy iNs did not affect p-tau accumulation (Additional file 1: Fig. S6a). Remarkably, thioflavin T-positive deposits in AD iNs were significantly increased by *APOE* ε4 induction at the amyloid-seeding stage (Fig. 2i, j). These results indicate that the induction of *APOE* ε4 at the amyloid early-seeding stage, but not at the aggregation stage, accelerates both tau phosphorylation and amyloid aggregation.

**Fig. 3:**
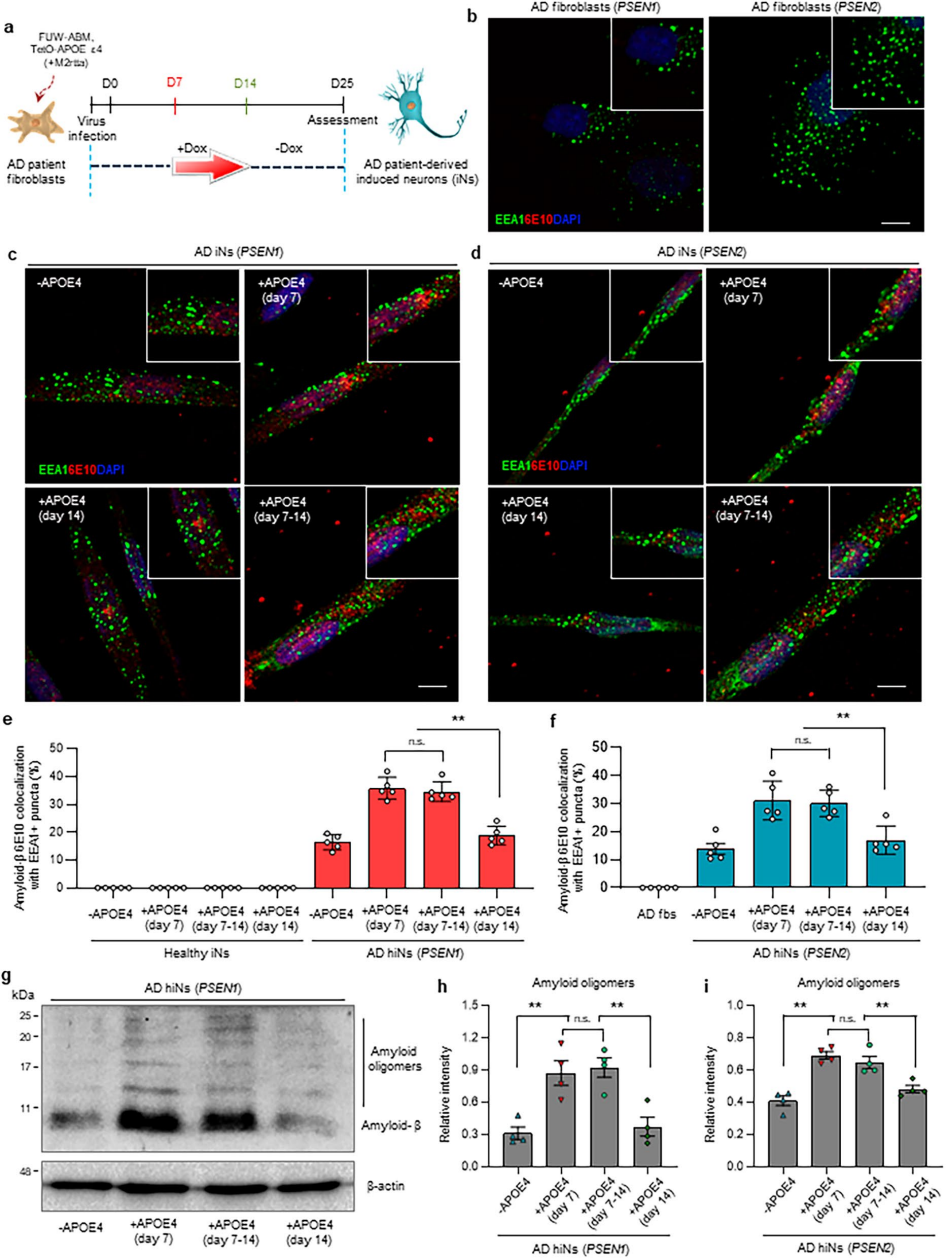
The effect of transient induction of *APOE* ε4 during the amyloid-seeding stage. **a** Schematic of the doxycycline-inducible system to partially express *APOE* ε4 in AD-patient-derived iNs. Doxycycline was added at the time-point of amyloid-seeding stage (day 7) and was removed on day 14. **b** Immunostaining of EEA1 and Aβ (6E10) in AD patient fibroblasts harboring *PSEN1* (left) or *PSEN2* (right) mutation. Scale bar, 10 µm. **c**, **d** Immunostaining of EEA1- and  Aβ (6E10) in *APOE* ε4-expressing AD patient iNs harboring *PSEN1* (**c**) or *PSEN2* (**d**) mutation. Doxycycline was withdrawn from the culture 7 days after the initial *APOE* ε4 induction. Scale bar, 10 µm. **e** Quantification of EEA1- and  Aβ-positive puncta in AD patient iNs harboring *PSEN1* mutation. Data represent mean ± SEM. ANOVA-test, **P < 0.01; *n* = 5 per sample. **f** Quantification of EEA1 and  Aβ-positive puncta in AD patient iNs harboring *PSEN2* mutation. Data represent mean ± SEM. ANOVA-test, **P < 0.01; *n* = 5 per sample. **g**, **h**, **i** Western blot analysis of Aβ (6E10) oligomers in *APOE* ε4-expressing AD patient iNs on day 25. Data represent mean ± SEM. ANOVA-test, *P < 0.05; *n* = 4 per sample. + APOE4 (day 7): AD patient iNs expressing *APOE* ε4 from day 7; + APOE4 (day 7–14): AD patient iNs expressing *APOE* ε4 during days 7–14; + APOE4 (day 14): AD patient iNs expressing *APOE* ε4 from day 14; -APOE4: AD patient iNs with no *APOE* ε4 expression

We next investigated the effect of transient induction of *APOE* ε4 during the amyloid-seeding stage. Doxycycline-mediated *APOE* ε4 induction could be maintained for 8 days only (days 7–14) after ABM induction, which resulted in the transient induction of APOE ε4 (Fig. 3a). Interestingly, we found that the increase in the number of amyloid puncta caused by *APOE* ε4 induction at the amyloid-seeding stage did not reduce even when *APOE* ε4 induction was withdrawn from AD iNs (Fig. 3b-d). Similarly, the number of EEA1- and Aβ-positive puncta was not affected by the withdrawal of *APOE* ε4 in AD patient iNs (Fig. 3e, f). Additionally, despite withdrawing doxycycline at day 14 of ABM induction, the accumulation of Aβ oligomers markedly increased in APOE ε4-expressing AD patient iNs (Fig. 3g–i, Additional file 1: Fig. S7a). However, the EEA1- and Aβ-positive puncta were not detected in healthy iNs overexpressing *APOE* ε4 (Additional file 1: Fig. S8a). These data suggest that the presence of *APOE* ε4 at the Aβ early-seeding stage is critical for aggravating sporadic AD pathogenesis.

### Molecular mechanism of *APOE* ε4-dependent AD phenotypes in AD iNs with *APOE* ε4 induction during the amyloid-seeding stage

**Fig. 4:**
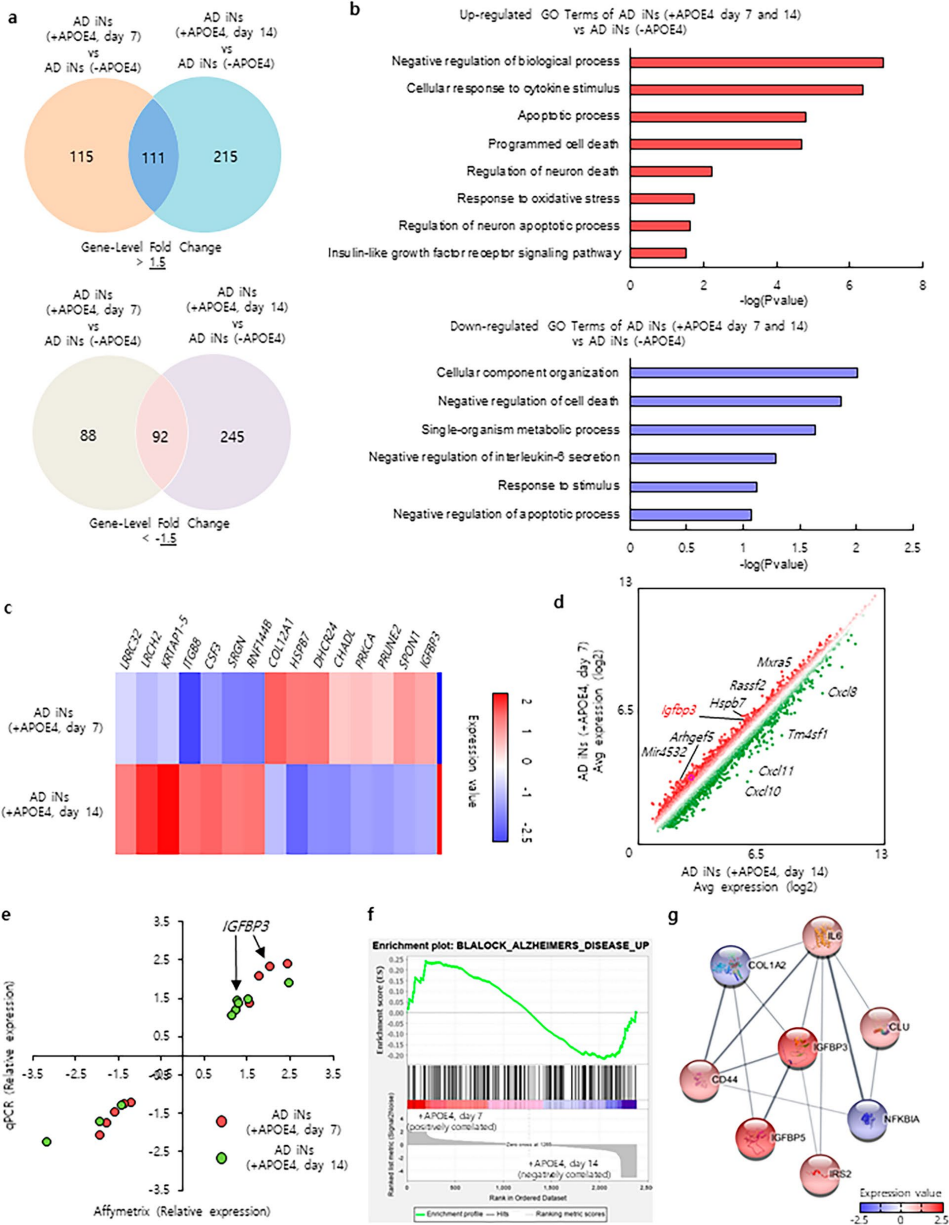
Global gene expression analysis of *APOE* ε4-expressing AD patient iNs during amyloid-seeding stage. **a** Venn diagram showing the overlap of differentially expressed genes between patient iNs expressing *APOE* ε4 from the amyloid-seeding stage and after the amyloid-seeding stage. The number of 1.5-fold upregulated (top) and 1.5-fold downregulated (bottom) genes are displayed on the Venn diagram. Top: *P*-value < 5.124e-136. Bottom: *P*-value < 2.960e-112. **b** Gene ontology analysis of the differentially expressed genes between *APOE* ε4-expressing AD patient iNs compared with that of AD patient iNs. **c** Heatmap showing microarray expression of differentially expressed genes in *APOE* ε4-expressing AD patient iNs at the amyloid-seeding stage or the amyloid progressive stage. **d** Scatter plot of the microarray data between *APOE* ε4-expressing patient iNs at the amyloid-seeding stage and *APOE* ε4-expressing AD patient iNs after the amyloid-seeding stage. **e** Scatterplot illustrating the concordance for 9 gene-expression values measured by microarray (x axis) and real-time qPCR (y axis) under each condition. **f** Gene set enrichment analysis of the microarray data from *APOE* ε4-expressing AD patient iNs at the amyloid-seeding stage and *APOE* ε4-expressing AD patient iNs after amyloid-seeding stage. **g** Graph of the IGFBP3 protein interaction network related to AD risk factors, which is subnetworked by containing direct interaction with proteins encoded by up-regulated overlapping genes (IGFBP5, CLU, IL6, CD44, IRS2) and down-regulated overlapping genes (COL1A2, NFKBIA) in *APOE* ε4-expressing AD patient iNs at the amyloid-seeding stage

To understand the molecular mechanism of *APOE* ε4-dependent AD pathologies in AD iNs with *APOE* ε4 induction at the Aβ early-seeding stage, we compared the global transcriptome profiles of AD patient iNs expressing *APOE* ε4 from the amyloid-seeding stage and from the amyloid-aggregation stage. *APOE* ε4-expressing AD patient iNs showed dramatic changes in the global gene expression compared to the control AD patient iNs (Fig. 4a). AD patient iNs expressing *APOE* ε4 from the amyloid-seeding stage displayed a significant amount of DEGs (FC ≥ 1.5), including 115 upregulated and 88 downregulated genes (Fig. 4a). To indicate a broader pathological pathway representing *APOE* ε4 effects, we performed gene ontology (GO) analysis. The *APOE* ε4-enriched GO terms and gene sets with *APOE* ε4-specific ontologies reflected apoptotic processes, neuronal death, and oxidative stress (Fig. 4b). Moreover, the upregulated genes, including *IGFBP3, IGFBP5*, and *BMP2*, are particularly related to the insulin-like growth receptor signaling pathway (Fig. 4c).

We next performed differential gene expression analyses by comparing iNs expressing *APOE* ε4 from the amyloid-seeding stage versus the amyloid-aggregation stage. We identified IGFBP3, a major binding protein of IGF-1, among the highly expressed genes in the amyloid-seeding stage (Fig. 4c, d). We also noted a close correlation between RT-PCR and microarray analyses for *IGFBP3* expression in AD iNs (Fig. 4e). In addition, we observed that the differential gene patterns of *APOE* ε4 expression at the amyloid-seeding stage were enriched in the brain gene expression of AD patients (Fig. 4f). Moreover, the protein–protein interaction network showed that IGFBP3 directly interacts with the enriched genes of AD patients and *APOE* ε4-expressing AD patient iNs at the Aβ early-seeding stage (Fig. 4g), indicating that IGFBP3 might functionally mediate the molecular pathology of sporadic AD with* APOE* ε4 expression at the Aβ early-seeding stage.

**Fig. 5:**
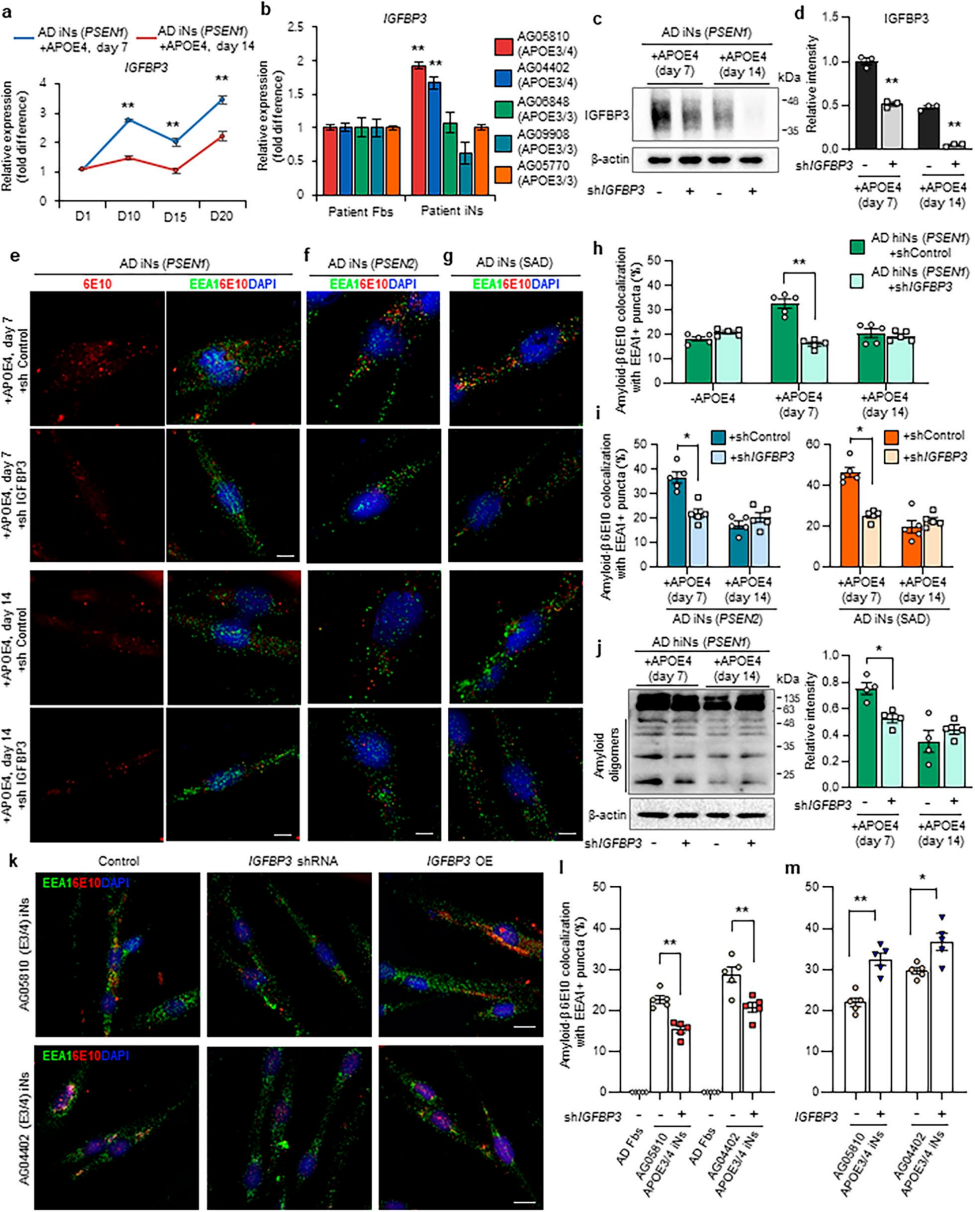
Knockdown of IGFBP3 decreases Aβ peptides in *APOE* ε4-expressing AD patient and *APOE* ε3/4 patient iNs at amyloid-seeding stage. **a** Validation of *IGFBP3* expression between *PSEN1-*harboring AD iNs expressing *APOE* ε4 from the amyloid-seeding stage and from the amyloid oligomer-progressive stage. Data represent mean ± SEM. ANOVA-test, **P < 0.01; *n* = 4 per sample. **b** Quantitative RT-PCR analysis of *IGFBP3* expression in AD patient iNs derived from *APOE* ε3/4 patient fibroblasts (AG05810 and AG04402), *PSEN1* patient fibroblasts (AG06848), *PSEN2* patient fibroblasts (AG09908), and sporadic AD patient fibroblasts (AG06869). Data represent mean ± SEM. ANOVA-test, **P < 0.01; *n* = 4 per sample. **c, d** Western blot analysis of IGFBP3 in AD-patient-derived iNs treated with *IGFBP3*-shRNA. Data represent mean ± SEM. ANOVA-test, **P < 0.01; *n* = 3 per sample. **e** Immunofluorescence of EEA1 and  Aβ (6E10) in AD patient (*PSEN1* mutation) iNs treated with *IGFBP3* knockdown. *APOE* ε4 expression was induced by doxycycline at amyloid-seeding or amyloid-progressive stage. Scale bars, 10 µm. **f, g** Immunofluorescence of EEA1 and  Aβ (6E10) in familial AD patient (**f**, *APOE* ε3/3 genotype) or sporadic AD patient (**g**, *APOE* ε3/3 genotype) cell line treated with *IGFBP3* knockdown. Scale bars, 10 µm. **h** Quantification of EEA1- and  Aβ-positive puncta in AD patient iNs harboring *PSEN1* mutation treated with *IGFBP3*-shRNA. Data represent mean ± SEM. ANOVA-test, **P < 0.01; *n* = 5 per sample. **i** Quantification of EEA1- and  Aβ-positive puncta in familial AD patient harboring *PSEN2* mutation (left, *APOE* ε3/3 genotype) or sporadic AD patient (right, APOE ε3/3 genotype) cell line treated with *IGFBP3* knockdown. Expression of *APOE* ε4 was induced by doxycycline at amyloid-seeding or amyloid-progressive stage. Data represent mean ± SEM. ANOVA-test, *P < 0.05; *n* = 5 per sample. **j** Western blot analysis shows the decrease of Aβ oligomers in *APOE* ε4-expressing AD patient iNs harboring *PSEN1* mutation treated with *IGFBP3*-shRNA. Data represent mean ± SEM. ANOVA-test, *P < 0.05; *n* = 4 per sample. **k** Immunostaining of EEA1 and  Aβ in AD patient iNs harboring *APOE *ε3/4. Knockdown or overexpression of *IGFBP3* was treated in the culture before the amyloid initial phase. Scale bar, 20 µm. **l, m** Quantification of EEA1- and  Aβ-positive puncta in AD patient iNs harboring *APOE* ε3/4 mutation. Data represent mean ± SEM. ANOVA-test, *P < 0.05, **P < 0.01; *n* = 5 per sample. + APOE4 (day 7): AD patient iNs expressing *APOE* ε4 from day 7; + APOE4 (day 14): AD patient iNs expressing *APOE* ε4 from day 14; -APOE4: AD patient iNs with no *APOE* ε4 expression

Next, we examined the functional role of IGFBP3 in AD iNs that expressed *APOE* ε4 from the amyloid-seeding stage. We initially confirmed the expression levels of *IGFBP3* using RT-PCR assay in the additional AD iNs. *IGFBP3* was highly expressed in the *APOE* ε4-expressing patient iNs (Fig. 5a, S9a). Remarkably, we found that *IGFBP3* was significantly elevated in additional sporadic AD patient-derived iNs with the *APOE* ε3/4 allele (Fig. 5b). However, we did not observe any differential expression of *IGFBP3* in AD patient-derived iNs with *APOE* ε3/3 allele (Fig. 5b), suggesting that the upregulated *IGFBP3* may be responsible for AD pathogenesis in *APOE* ε4-dependent sporadic AD patient iNs.

To determine whether IGFBP3 functionally contributes to *APOE* ε4-associated AD phenotypes during Aβ-seeding stage, we generated shRNA lentivirus against *IGFBP3* and initially confirmed the differential expression of IGFBP3 through RT-PCR and Western blot analysis (Fig. 5c, d; Fig. S9b). Upon *IGFBP3* knockdown by shRNA, the increase in Aβ puncta caused by *APO*E ε4 expression at the amyloid-seeding stage was significantly reduced in AD patient iNs (Fig. 5e, h). However, the number of Aβ puncta did not change in AD iNs with *APOE* ε4 expression after amyloid-seeding stages upon *IGFBP3* knockdown (Fig. 5e, h). Similarly, *IGFBP3* knockdown significantly reduced the number of Aβ puncta in AD patient iNs harboring *PSEN2* mutation and *APOE* ε3/3 allele sporadic AD patient iNs (Fig. 5f, g, i). Additionally, Western blotting showed that *IGFBP3* inhibition reduced the accumulation of Aβ polymers in patient iNs expressing *APOE* ε4 from the amyloid-seeding stage (Fig. 5j, Additional file 1: Fig. S9c). We also observed that the increased p-tau accumulation was significantly reduced in AD patient iNs harboring *PSEN1* mutation upon *IGFBP3* knockdown (Additional file 1: Fig. S10a, b). These data support IGFBP3 mediation of progressive amyloidogenesis and tau pathology induced by *APOE* ε4 at Aβ-seeding stages in sporadic AD.

Finally, we examined the functional effects of IGFBP3 in *APOE* ε3/4 patient iNs. We previously detected an increase in *IGFBP3* expression in sporadic AD patient iNs with the *APOE* ε3/4 genotype (Fig. 5b). Furthermore, the elevated Aβ puncta in *APOE* ε3/4 patient iNs were significantly decreased upon *IGFBP3* knockdown (Fig. 5k, l). We also observed a significant increase in Aβ puncta in sporadic AD patient iNs harboring *APOE* ε3/4 upon *IGFBP3* overexpression (Fig. 5k, m). These results suggest that IGFBP3 functionally mediates the *APOE* ε3/4-dependent AD pathologies during the amyloid-seeding state.

## Discussion

Carrying the ε4 allele of *APOE* is a major genetic risk factor for late-onset AD. APOE ε4 modulates the accumulation and subsequent deposition of Aβ peptides in the brain. Aβ aggregation has been identified as the earliest detectable pathology in the brains of humans and transgenic mice, before the formation of plaques in AD. A number of previous studies have indicated that once the Aβ seeding and nucleation occur, apoE begins to play a critical role in the initial formation of Aβ aggregation; however, it is not much relevant during the growth period of plaques [12, 13]. In addition, it is still unclear how *APOE* ε4 drives such effects during different stages of amyloid development in human neurons.

A previous study has reported that endogenously expressed *APOE* ε4 enhances Aβ production and GABAergic neuron degeneration in cultured human neurons [25]. Additionally, we demonstrated that iNs derived from the fibroblasts of *APOE* ε4 AD-patients could be used as a tool to identify the pathogenic mechanisms of sporadic AD [18]. Another study has shown that astrocytic apoE4 during the initial seeding stage affects dystrophic neurites around plaques and impairs Aβ clearance in apoE-inducible mouse models [12]. Conversely, reducing apoE4 prior to amyloid deposition greatly affects changes in the plaque load and plaque properties as opposed to the effects of reducing apoE4 once Aβ aggregation has already begun in mouse models [13, 26]. Our study provides the first evidence that expression of *APOE* ε4 in AD patient-derived iNs at the amyloid-seeding stage is sufficient to facilitate amyloid pathogenesis. Specifically, transient expression of *APOE* ε4 during the amyloid-seeding stage (days 7–14 of reprogramming) still increased Aβ oligomers and amyloidosis, suggesting that *APOE* ε4 has the greatest impact during the early phase of Aβ development in AD-patient-derived neurons.

Under normal physiological conditions, APOE is mainly expressed by astrocytes [27, 28]. Under diseased conditions, the disease-associated microglia (DAM) also express high levels of APOE [29]. Neurons typically only express APOE under stress conditions [30, 31]. Thus, although APOE was not detectable in different stages of control and AD iNs without doxycycline treatment in our experiments (data not shown), we can reason that iNs that have undergone dramatic cell fate changes are in more stressful conditions, and it is most likely that these conditions increase the sensitivity to APOE in iNs compared to normal neurons in the brain. From this point of view, it is difficult to accurately describe the AD situation that occurs in the aged brains through *APOE* ε4-expressing iNs. Thus, in order to show the effects of *APOE* ε4 iNs more accurately, additional studies are needed to examine the effects of *APOE* ε4 in iNs under the stress conditions caused by co-culture with AD astrocytes and microglia. Taken together, the present study clearly showed the development of sporadic AD in the presence of *APOE* ε4 at the amyloid early-seeding stage in the iNs, highlighting the role of neuronal *APOE* ε4 under stress conditions in AD development in the brain.

It has recently been reported that neuronal LRP1, a major apoE receptor, mediates increased Aβ deposition and disruption of Aβ clearance depending on the *APOE* genotype in the brains of patients with AD [32–34]. Herein, to identify the functional mechanisms of *APOE* ε4-mediated AD pathologies during the initial amyloid-seeding stage, we examined the DEGs in AD patient-derived iNs at different amyloid stages. Interestingly, we detected an increase in *IGFBP3* expression in AD patient iNs expressing *APOE* ε4 from the initial phase of Aβ development. We also observed that the IGFBP3 complex is closely linked to *APOE* ε4-induced AD phenotypes during the initial formation of Aβ aggregation in AD patient iNs. A previous study reported that IGFBP3 is released from astrocytes after Aβ42 stimulation, leading to Aβ aggregation and tau phosphorylation in neurons [35]. However, the functional connection between IGFBP3 and *APOE* ε4 in AD patient neurons was not previously known. Moreover, sporadic AD patient iNs harboring *APOE* ε3/4 exhibited a significantly higher *IGFBP3* expression than that observed in AD patient-derived iN lines with the *APOE* ε3/3 genotype. More importantly, we demonstrated that the knockdown of *IGFBP3* efficiently suppressed *APOE* ε4-dependent AD pathologies during amyloid early-seeding stage in sporadic AD patient iNs, suggesting the role of IGFBP3 in *APOE* ε4-induced AD phenotypes.

## Conclusions

The present study implies a functional connection between the genetic loci and sporadic AD and provides a critical insight into the pathogenesis of *APOE* ε4-associated AD development. The iNs could serve as a human cellular platform to develop personalized medicine for more effective treatments of sporadic AD.

## Supplementary Information

**Additional file 1**. Supplementary figures.

## Data Availability

The authors declare that data supporting the findings of this study are available within the article and its Supplementary Information files or from the corresponding author on request.

## Abbreviations

AD: Alzheimer’s disease
iN: Induced neuron
APOE: Apolipoprotein E
PSEN1: Presenilin 1
PSEN2: Presenilin 2
IGFBP3: Insulin like growth factor binding protein 3
APP: Amyloid precursor protein
IGF-1: Insulin like growth factor-1
GO: Gene ontology
LRP1: LDL receptor related protein 1
