# Supplementary material for: APOE ε4-dependent effects on the early amyloid pathology in induced neurons of patients with Alzheimer’s disease
Source: Transl Neurodegener. 2022 Oct 25;11:45. doi: 10.1186/s40035-022-00319-9 (PMC9594913; doi:10.1186/s40035-022-00319-9)
Supplement: Supplementary file 1 — Additional file 1. Supplementary figures. [file 40035_2022_319_MOESM1_ESM.docx]

**APOE ε4-dependent effects on the early amyloid pathology in induced neurons of patients with Alzheimer’s disease**

Hongwon Kim^1,2,#^, Siyoung Kim^2,#^, Byounggook Cho^2^, Jaein Shin^2^, and Jongpil Kim^1,2*^

**Supplementary Information.**

**
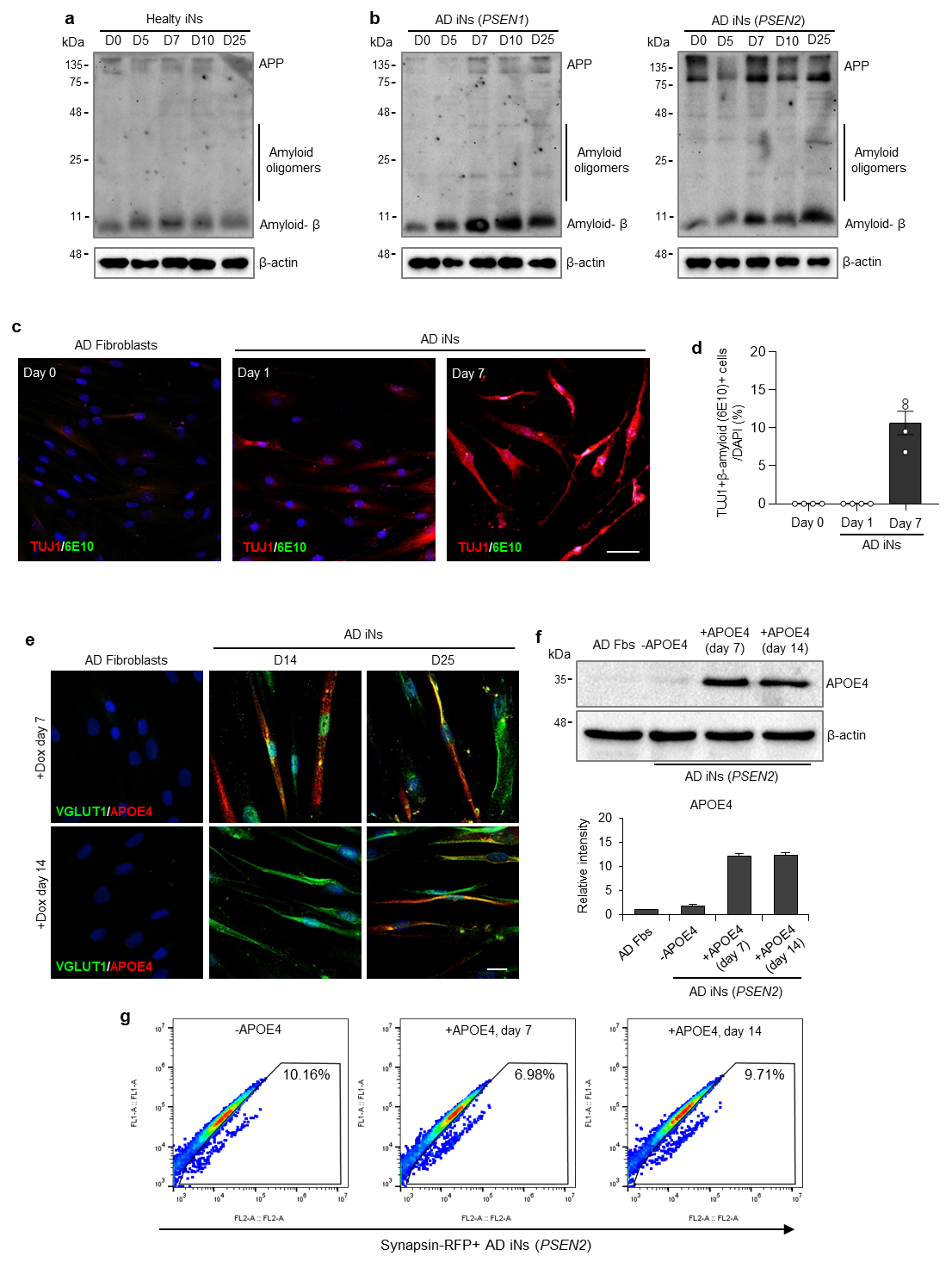
**

**Supplemental Figure 1. a, b** Western blot analysis presents the level of amyloid-β (6E10) oligomers in the iNs of healthy (**a**) and AD patients (**b**) at different time points. **c** Immunofluorescence for TUJ1 and amyloid-β (6E10) in AD-patient-derived iNs at day 0, day 1, and day 7. Scale bar, 50 µm. **d** Quantification of TUJ1- and amyloid-β (6E10)-positive cells in AD-patient-derived iNs. Data represent mean±SEM. *n* = 4 per sample. **e** Immunofluorescence for VGLUT1 and APOE ε4 in AD-patient-derived iNs at day 0, day 14, and day 25. Scale bar, 20 µm. **f** Levels of APOE ε4 and β-actin in AD-patient-derived iNs harboring *PSEN2* mutation treated with doxycycline at day 7 or 14. Data represent mean±SEM; *n* = 3 per sample. **g** Fluorescence-activated cell sorting analysis of Synapsin–RFP-positive cells from *PSEN2* mutant AD-patient cell line at different amyloid stages.
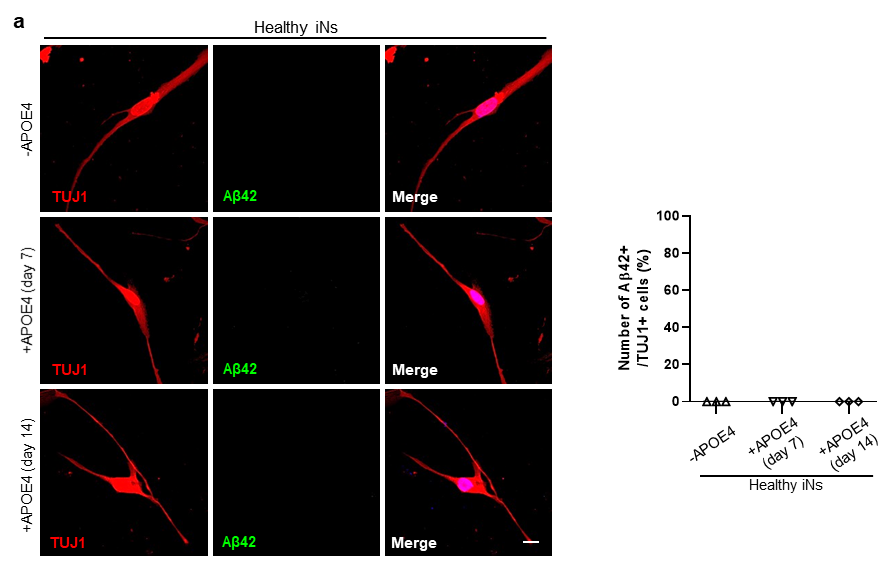


**Supplemental Figure 2. a** Representative immunofluorescence images of amyloid-β 42-positive iNs at day 25. To express APOE ε4 in healthy control iNs, doxycycline was treated on day 7 or 14. Scale bar, 20 µm.


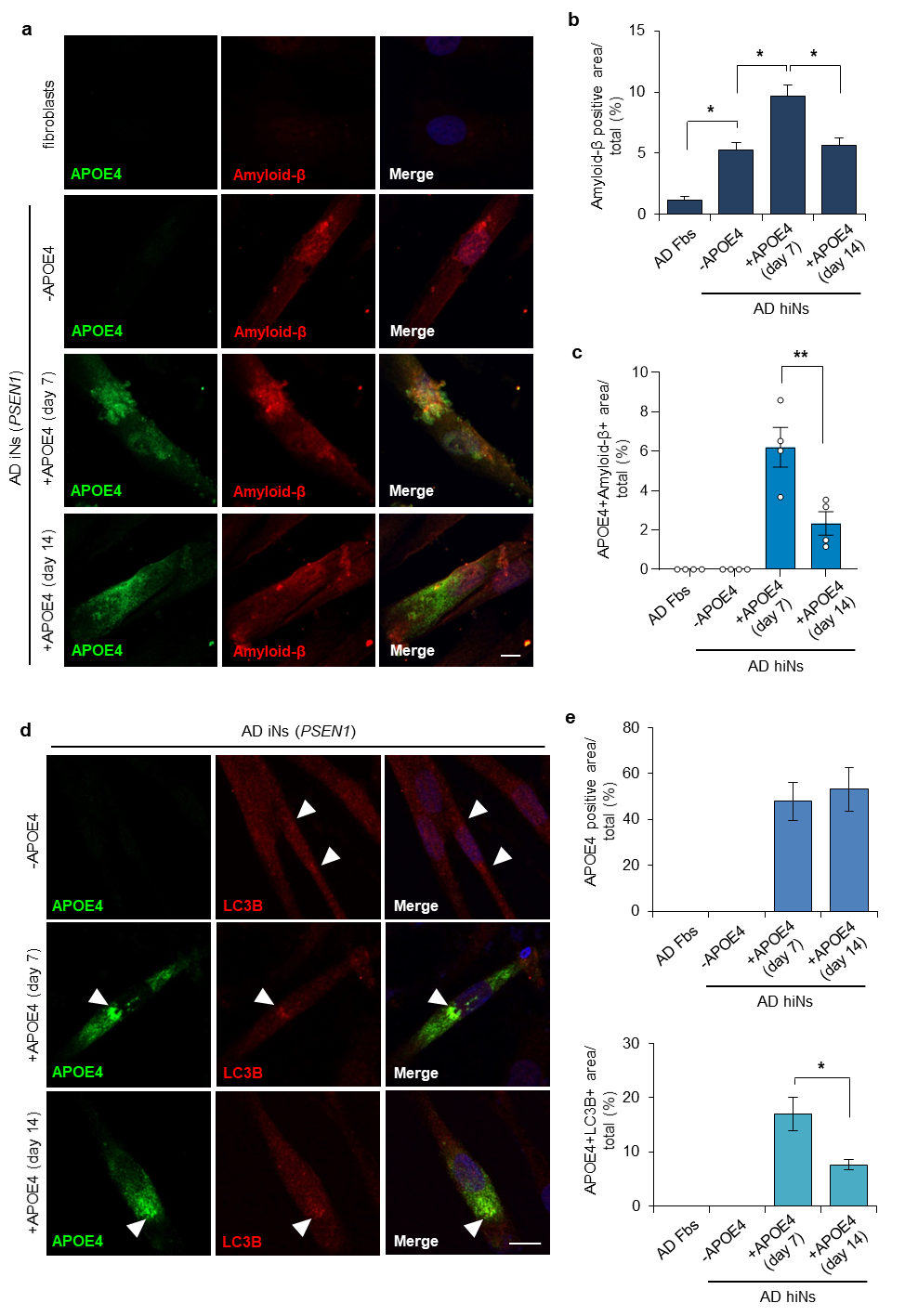


**Supplemental Figure 3. a** Representative immunofluorescence images of APOE ε4 and Aβ c-terminal-positive cells for AD-patient (*PSEN1* mutation) cell line. Scale bar, 10 µm. **b** Measurement of APOE ε4-positive area compared to the total area in APOE ε4-expressing AD-patient iNs treated with doxycycline at day 7 or 14. Data represent mean±SEM. *ANOVA-test*, **P* < 0.05; *n* = 3 per sample. **c** Measurement of co-localized area compared to the total immunofluorescent area in APOE ε4-expressing AD-patient iNs. Data represent mean±SEM. *ANOVA-test*, ***P* < 0.01; *n* = 4 per sample. **d** Representative immunofluorescence images of APOE ε4 and LC3B-positive cells for AD-patient (*PSEN1* mutation) cell line. Scale bar = 20 µm. **e** Measurement of APOE ε4+ area (top) and APOE ε4+, LC3B+ area (bottom) compared to the total area in APOE ε4-expressing AD-patient iNs treated with doxycycline at day 7 or 14. Data represent mean±SEM. *ANOVA-test*, **P* < 0.05*; n* = 5 per sample.

**
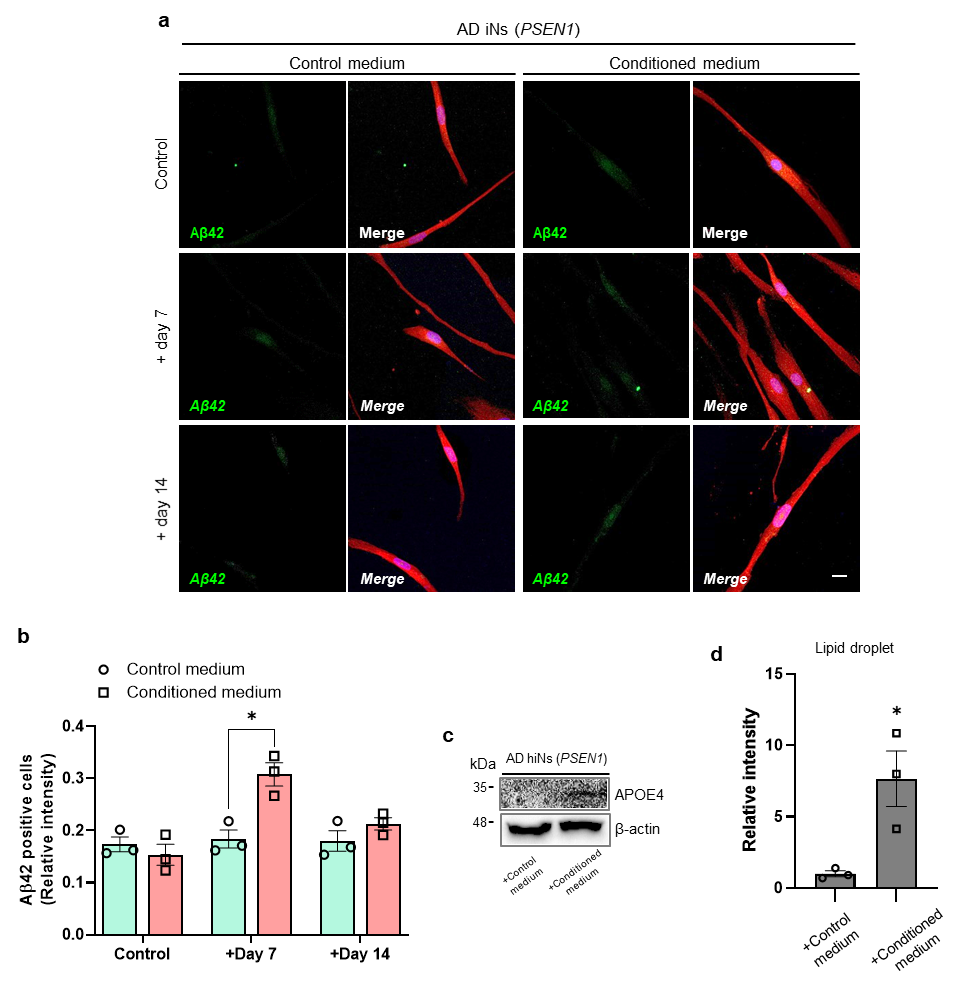
**

**Supplemental Figure 4. a** Representative immunofluorescence images of TUJ1- and Aβ42-positive cells for AD-patient (*PSEN1* mutation) cell line. APOE ε3 lipid particles are contained in the control medium. APOE ε4 lipid particles are contained in the conditioned medium. Scale bar, 20 µm. **b** Quantification of Aβ42-positive cells in AD-patient-derived iNs treated with APOE lipid particles at different amyloid stages. Data represent mean±SEM. ANOVA-test, *P < 0.05; n = 3 per sample. **c** Levels of APOE ε4 and β-actin in AD-patient-derived iNs treated with APOE ε3 or APOE ε4 lipid particles. **d** Measurement of lipid droplet in AD-patient iNs treated with APOE ε3 or APOE ε4 lipid particles. Data represent mean±SEM. *ANOVA-test*, **P* < 0.05; *n* = 3 per sample.


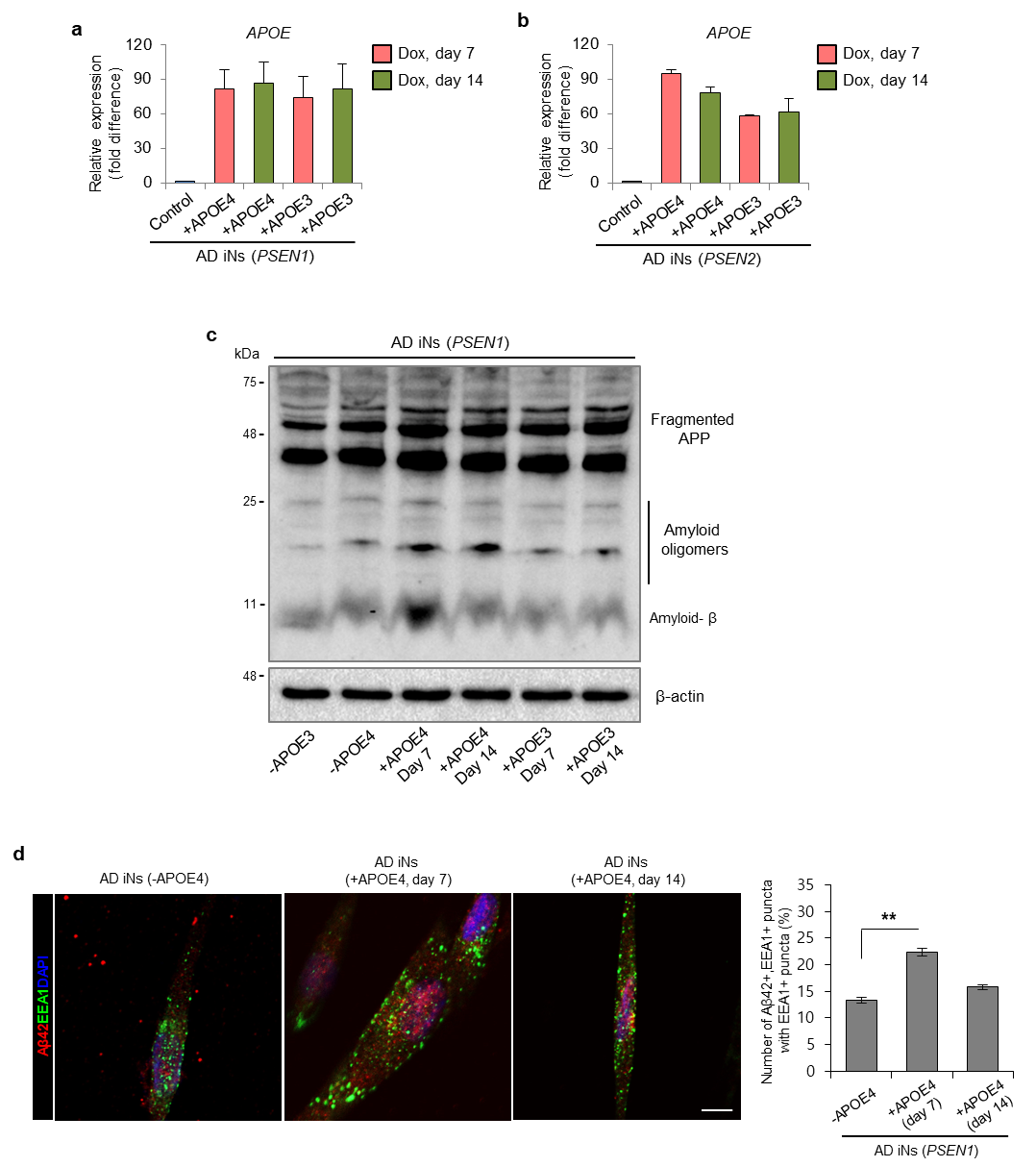


**Supplemental Figure 5. a, b** Validation of *APOE* expression in APOE ε4- or APOE ε3-expressing AD-patient iNs derived from familial AD patients with *PSEN1* (**a**) or *PSEN2* (**b**) mutation. Data represent mean±SEM. *ANOVA-test; n* = 3 per sample. **c** Western blotting of APOE ε4- or APOE ε3-expressing AD-patient iNs derived from familial AD patients with *PSEN1* mutation treated with doxycycline at day 7 or 14. **d** Representative image of Aβ42 and EEA1 in APOE ε4-expressing AD-patient iNs at different amyloid stages. Data represent mean±SEM. ANOVA-test, **P < 0.01; n = 3 per sample.

**
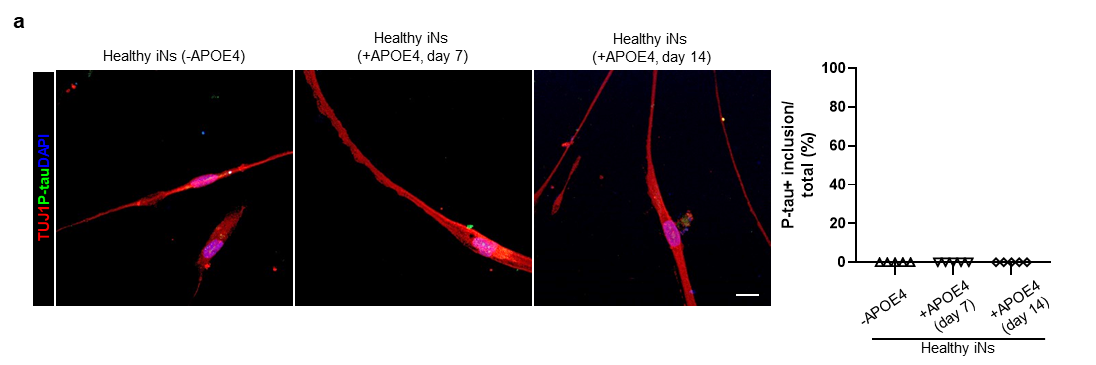
**

**Supplemental Figure 6. a** Representative images of hyperphosphorylated tau in healthy control iNs on day 25. Scale bar, 20 µm.

**
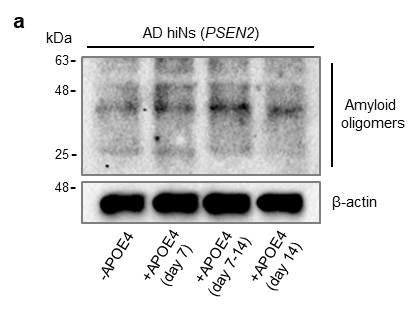
**

**Supplemental Figure 7. a** Western blotting of β-amyloid (6E10) oligomers in APOE ε4-expressing AD-patient iNs harboring *PSEN2* mutation on day 25.

**
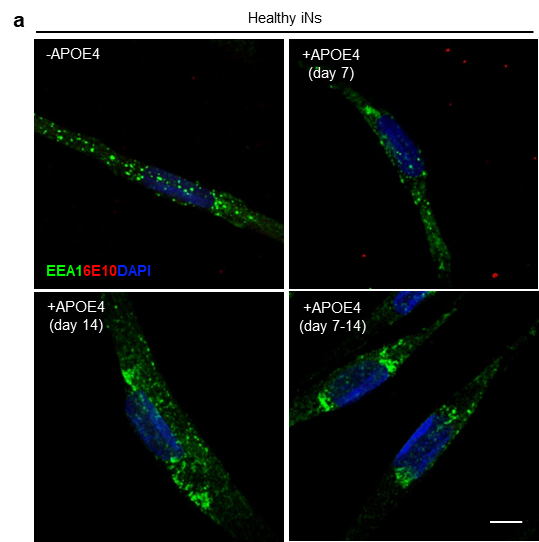
**

**Supplemental Figure 8. a** Immunostaining of EEA1 and amyloid-β (6E10) in healthy control iNs. Doxycycline was withdrawn from the culture 7 days after the initial APOE ε4 induction. Scale bar, 10 µm.


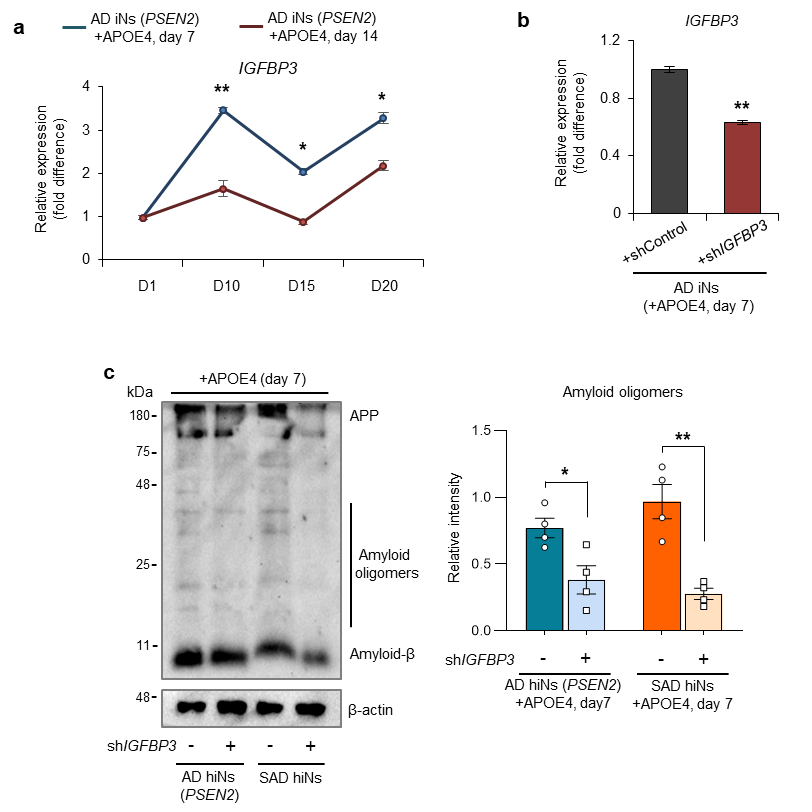


**Supplemental Figure 9. a** Validation of *IGFBP3* expression between amyloid-seeding stage and amyloid oligomer-progressive stage at different time points in AD-patient cell harboring *PSEN2*. Data represent mean±SEM. *ANOVA-test*, **P* < 0.05, ***P* < 0.01; *n* = 4 per sample. **b** Relative expression of *IGFBP3* gene in APOE ε4-expressing AD-patient iNs treated with *IGFBP3*-shRNA. Data represent mean±SEM. *ANOVA-test*, ***P* < 0.01; *n* = 4 per sample. **c** Western blot analysis presents the level of amyloid-β (6E10) oligomers in the iNs of AD patients harboring *PSEN2* mutation and sporadic AD patients. Data represent mean±SEM. *ANOVA-test*, **P* < 0.05, ***P* < 0.01; *n* = 4 per sample.


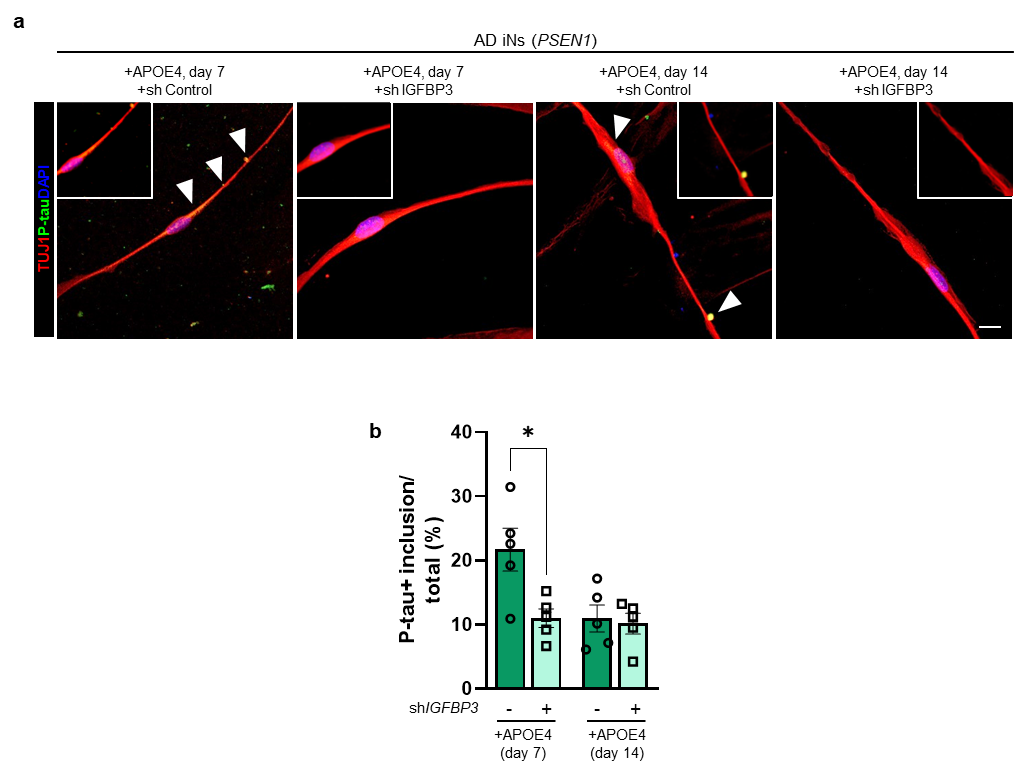


**Supplemental Figure 10. a** Representative immunofluorescence images of hyperphosphorylated tau in APOE ε4-expressing AD-patient iNs treated with *IGFBP3*-shRNA. Scale bar, 20 µm. **b** Measurement of phospho-tau-positive inclusions compared to the total area at different amyloid stages. Knockdown of *IGFBP3* was treated in the culture before the amyloid initial phase. Data represent mean±SEM. *ANOVA-test*, **P* < 0.05; *n* = 5 per sample.
